# Organizing ourselves for a conscious use of Artificial Intelligence in Scientific Publications: human responsibility as guiding principle

**DOI:** 10.17843/rpmesp.2026.433.17075

**Published:** 2026-08-31

**Authors:** Lely Solari

**Affiliations:** 1 Instituto Nacional de Salud, Lima, Peru. Instituto Nacional de Salud Lima Peru

One of the most characteristic elements of human nature is the constant search for new technologies to change the world. The history of mankind has been shaped by a series of technological innovations that, having been many times developed by only a handful of people, have had enormous repercussions at a global level and throughout the years. Some of them have been material technologies, like the gunpowder from China, which was initially configured to marvel people in light shows, but later contributed to wipe out whole populations. Or like the penicillin, that almost after a century of use continues saving lives, but used in allergic persons can cause death. Most technologies are not neutral; it is the responsibility of the use to give them a positive utility. In this line, Artificial Intelligence (AI) is a technology that, despite being less tangible and having been created recently, already has an enormous impact in humanity and there is wide consensus on it being one of the determinants of our future as a species.

AI consists of the engineering of systems, machines and programs (software) that can imitate the human intelligence and even its behavior, being thus able to fulfill intellectual tasks with great precision and scope. Unlike automatization, that is about informatic programs designed with a series of rules that accomplish tasks according to premises that are provided to them (for instance, to generate an email as part of a procedure), many AI tools, in particular generative AIs, are capable of generating new ideas, to put them into text or images and even of taking decisions ^1^. Notwithstanding, it is crucial to note that, as opposed to the human beings, they are not capable of taking responsibility or their actions, including these decisions they can take, nor of their potential repercussions or impact.

In the case of scientific publications, this last point is critical. Scientific publication is the existing way through which the people that develop knowledge using the scientific method can communicate it, following a series of methodological and ethical standards that the scientific community has been building and formalizing through their respective institutions throughout the years. To take responsibility for the published content is key to scientific rigor, as the publication is directly linked to the author and thus to his (her, their) credibility, image and even honor. The author can err, moreover, science evolves and paradigms change. However, what cannot happen, is that the author does not provide information in a transparent way about all the tools (including AI) and procedures used, or that he (she, they) manipulates the results that were reached or intentionally bias their interpretation. Nor is it acceptable that the author, after publishing, disavows himself of the final content of the publication, or even reinterprets what he (she, they) wrote, turning it into something different.

It is based on this premise that the most important institutions guiding good practices and ethics in scientific publication are working to offer guidelines and recommendations about how the medical journals can manage the use of AI, not only concerning the authors and the reviewers, but also for their own editorial processes. Many AI tools area available, and many of them can ease a wide range of processes and aid authors, reviewers and the journals, but as with the gunpowder and the penicillin, it is the human who has to define its utility, according to his (her, their) priorities, and to take responsibility for the correct use in all aspects, and for the consequences generated by this use.

Accordingly, the Editor Committee of the Revista Peruana de Medicina Experimental y Salud Pública, official platform of scientific communication of the Instituto Nacional de Salud, has reviewed the guidelines and recommendations-even the ones still in development- of the global institutions leading the way in this issue, including the International Committee of Medical Journal Editors (ICMJE) ^2^, the Committee on Publications Ethics (COPE) ^3^^,^^4^ and the World Association of Medical Editors (WAME) ^5^. Then, it has selected the key elements to be discussed in order to update its editorial policy for the use of AI in scientific publications, classifying the scenarios where it is allowed and where it is not, for authors, reviewers and the journal itself (https://rpmesp.ins.gob.pe/index.php/rpmesp/aipolicy).

Noteworthy, the approach we have given to this discussion is totally detached from the interest or rejection that this issue can generate individually. Currently, there is available technology that can support editorial work and useful tools for the researchers to develop their projects and produce the manuscripts that communicate their results, and many of them are already using it, although they do not always declare it. It is necessary that the authors understand that to disclose AI use is mandatory and that they have to be totally transparent in this sense. For this, scientific journals are the ones that have to clearly establish the guidelines of where and how to report the use of AI, until, in the near future, we have standardized ways in which to make these disclosures ^6^.

Currently, there is useful AI technology that medical journals can use to evaluate the presence of duplication and/or plagiarism, to assess the adequate use of citations and references, to find ad-how reviewers, among other tasks. Technologies that are not connected to the cloud and can ensure confidentiality in the handling of the information included in articles that are not yet published can be used for such tasks. On the other hand, AI technologies that are attached to centralized servers, where uploaded manuscripts can be reused by the owners of those AI technologies, should be avoided. Medical journals must honor the trust that authors put in their processes when sending their papers, which opposes to sharing them with AI technologies that do not disclose the path that the information that is uploaded to their systems undertakes. Finally, both authors as editors have to assume the whole responsibility for the implications of the use of AI in their processes, be it scientific or editorial. 

It is well known that scientific and publication malpractices already existed long before the emergence of AI, but they can increase with the use of these technologies. For instance, they can be used to multiply manuscripts or citations, and even data ^7^. On the other hand, they can generate negative effects concerning equity in scientific publications, as it will be the groups with more access to AI technologies that do not share the information with the cloud (which require subscriptions) who will benefit the most ^8^. However, we can still take the chance to use AI tools to accomplish our priorities, establishing as the primary goal the production of useful and trustworthy knowledge for the development of our society and health.

Personally, there are some elements that make me keep the hope in believing it is possible to achieve a healthy governance in this issue of using AI technologies for scientific publications. When discussing the journal´s editorial policy I have proudly noticed that it was the youngest members if the editor committee who most strongly defended the human participation in the processes of writing the manuscripts, reviewing and editing them. Additionally, when defining the editorial policy, we have permanently adopted the principle of *primum non nocere*, trying no to lose whatever progress—however great or small—has been achieved in the country's scientific advancement, for the sake of a technology that some consider to offer, at most, a marginal contribution ^9^. Ultimately, as with any technology, it will be us humans who will determine its correct and effective use—at least for as long as the decision remains ours.
