# Supplementary material for: Organizing ourselves for a conscious use of Artificial Intelligence in Scientific Publications: human responsibility as guiding principle
Source: Rev Peru Med Exp Salud Publica. 2026 Aug 31;43(3):296–7. doi: 10.17843/rpmesp.2026.433.17075 (PMC13718566; doi:10.17843/rpmesp.2026.433.17075)
Supplement: Translation

# Translation: Organizándonos para un uso consciente de la inteligencia artificial en la publicación científica: la responsabilidad humana como eje rector

**Authors:** Lely Solari

**Affiliations:** 1 Instituto Nacional de Salud, Lima, Perú

Uno de los elementos más característicos del ser humano es la constante búsqueda de nuevas tecnologías para cambiar el mundo. La historia del hombre ha sido moldeada por una serie de innovaciones tecnológicas que, habiendo sido creadas muchas veces por un puñado de personas, han tenido enormes repercusiones a nivel global y a lo largo de la historia. Algunas de ellas fueron tecnologías materiales, como la pólvora proveniente de China, que inicialmente fue configurada para maravillar a las personas con espectáculos de luces, pero posteriormente contribuyó a exterminar a poblaciones enteras, o como la penicilina, que a casi 100 años de uso sigue salvando miles de vidas, pero utilizada en personas alérgicas puede llevar a la muerte. La mayoría de tecnologías no son neutras, es responsabilidad de quien las utiliza darles una finalidad positiva. En este sentido, la Inteligencia Artificial (IA), es una tecnología que a pesar de ser mucho menos tangible y, de haber sido creada recientemente, ya tiene un enorme impacto en la humanidad y existe amplio consenso en que va a ser un elemento determinante en nuestro futuro.

La IA consiste en la ingeniería de sistemas, máquinas y programas (software) que pueden imitar la inteligencia y hasta el comportamiento humano, logrando cumplir tareas intelectuales con alta precisión y alcance. A diferencia de la automatización, que se trata de programas informáticos diseñados con una serie de reglas que cumplen tareas de acuerdo a premisas que se les brinda (por ejemplo, generar un correo electrónico como parte de un procedimiento), diversas herramientas de IA, en particular las IA generativas, son capaces de generar ideas nuevas, plasmarlas en texto e imágenes, e incluso tomar decisiones ^1^. Sin embargo, es crucial notar que, a diferencia del ser humano, de lo que no son capaces es de responsabilizarse de sus acciones, incluyendo las decisiones que toman, ni de las potenciales repercusiones o impacto de las mismas.

En el caso de las publicaciones científicas, este último punto es crítico. Las publicaciones científicas son el medio existente para que las personas que desarrollan conocimiento utilizando el método científico, lo difundan, siguiendo una serie de estándares metodológicos y éticos que la comunidad científica ha ido construyendo a lo largo de los años y que ha formalizado a través de las instituciones que la representan. Responsabilizarse por el contenido difundido es justamente la clave del rigor científico, ya que la publicación está directamente ligada al autor, y por ende a su credibilidad, imagen e incluso honra. El autor puede equivocarse, además, la ciencia evoluciona y los paradigmas cambian. Pero lo que no puede ocurrir es que el autor no brinde información de manera transparente sobre todas las herramientas (incluyendo la IA) y procedimientos que usó, que cambie los resultados a los que llegó o que sesgue intencionalmente su interpretación. Tampoco es aceptable que el autor, luego de hacer su publicación, se desentienda del contenido de su producto final, o incluso que reinterprete lo que escribió, convirtiéndolo en algo distinto.

Es sobre esta premisa que las instituciones más importantes en el ámbito de las buenas prácticas y ética en publicación científica están trabajando para brindar guías y recomendaciones sobre cómo las revistas científicas deben gestionar el uso de la IA, no solamente por parte de los autores y los revisores, sino también dentro de sus propios procesos editoriales. Hay en el mercado muchas herramientas de IA que definitivamente pueden facilitar una amplia gama de procesos para los autores, los revisores y las revistas, pero, así como en el caso de la pólvora o la penicilina, es el ser humano el que debe definir el uso que le da, de acuerdo a sus prioridades, y responsabilizarse de que este uso sea correcto en todos sus aspectos, así como de las consecuencias que se generen.

En este sentido, el Comité Editor de la Revista Peruana de Medicina Experimental y Salud Pública, órgano oficial de comunicación científica del Instituto Nacional de Salud del Perú, ha revisado los lineamientos y recomendaciones -incluso las que se encuentran en construcción- de las instituciones que brindan la pauta en este ámbito a nivel global, incluyendo el Comité Internacional de Editores de Revistas Médicas (ICMJE)^2^, el Comité de Ética en las Publicaciones (COPE)^3^^,^^4^^)^ y la Asociación Mundial de Editores Médicos (WAME)^5^. Posteriormente, ha seleccionado los elementos que considera clave, y los ha discutido en conjunto, para poder actualizar su política editorial de uso de la IA en la publicación científica, clasificando los ámbitos en los que está permitido y en los que no, tanto para los autores y revisores como para los editores, y la revista misma (https://rpmesp.ins.gob.pe/index.php/rpmesp/aipolicy).

Cabe resaltar que el abordaje que le hemos dado al uso de la IA no tiene que ver con el interés o rechazo que este tema nos pueda despertar de manera individual. En la actualidad, existe tecnología que puede brindar apoyo y herramientas útiles para que los investigadores desarrollen sus estudios y produzcan los manuscritos que los difunden, y muchos de ellos ya las están utilizando, aunque no necesariamente declarándolo. Es necesario que los autores entiendan que hacer esa declaración es una obligación y que deben ser totalmente transparentes en ese sentido. Para ello, son las revistas científicas quienes debemos establecer claramente la pauta sobre cómo y cuándo hacerlo, hasta que tengamos, en el futuro cercano, maneras estandarizadas de hacer estas declaraciones ^6^.

Actualmente también existe tecnología de IA útil que las revistas pueden usar para evaluar la presencia de duplicación y/o plagio, la corrección de las citas y referencias, para buscar revisores ad-hoc, entre otras tareas. Las tecnologías que no estén conectadas a la nube y aseguren la confidencialidad en el manejo de la información contenida en artículos que aún no están publicados, podrían utilizarse con estos fines. Por el contrario, las tecnologías de IA que utilizan servidores centralizados, donde los manuscritos compartidos pueden ser reutilizados por los gestores de dichas tecnologías, deben evitarse. Las revistas deben honrar la confianza que los autores ponen en sus procesos al enviar sus manuscritos, lo cual se contrapone a compartirlos con tecnologías de IA que no revelan a dónde irá a parar la información que se les comparte. Finalmente, tanto autores como editores tendrán que asumir la responsabilidad plena por las implicancias del uso de la IA en sus procesos, tanto científicos como de gestión.

Resulta innegable que las malas prácticas científicas y en publicación que ya existían antes de la aparición de la IA pueden incrementarse con el uso de estas tecnologías de IA. Por ejemplo, se pueden usar para multiplicar manuscritos o citas, y en el peor de los casos, datos ^7^. Por otro lado, pueden generar repercusiones negativas en el ámbito de la equidad en publicación científica, ya que serán los grupos con más acceso a las tecnologías de IA que no comparten la información con la nube (que requieren de un pago específico), los que más se beneficiarán de las mismas ^8^. Sin embargo, todavía podemos aprovechar la oportunidad de utilizar las herramientas de IA para el cumplimiento de nuestras prioridades, estableciendo como norte la producción de conocimiento científico útil y confiable para el desarrollo de la sociedad y su salud.

En lo personal, hay algunos elementos que me hacen mantener la esperanza en la posibilidad de lograr una gobernanza aceptable en el tema del uso de las tecnologías de IA en la publicación científica. Al discutir la política editorial, he visto con orgullo que los miembros más jóvenes del Comité Editor de la RPMESP han sido los que más han defendido la participación del humano en el proceso de redacción del manuscrito, la edición y la publicación del mismo. Además, en la construcción de la política editorial, en todo momento hemos recurrido al principio de *primum non nocere,* tratando de no perder lo mucho o poco que se ha logrado en el avance de la ciencia en el país, por la promesa de una tecnología que algunos consideran que tiene un aporte a lo sumo, marginal ^9^. En última instancia, como toda tecnología, seremos los humanos quienes determinaremos su uso correcto y efectivo, por lo menos, mientras todavía sea nuestra decisión.
